# Graphene–polymer coating for the realization of strain sensors

**DOI:** 10.3762/bjnano.8.3

**Published:** 2017-01-03

**Authors:** Carmela Bonavolontà, Carla Aramo, Massimo Valentino, Giampiero Pepe, Sergio De Nicola, Gianfranco Carotenuto, Angela Longo, Mariano Palomba, Simone Boccardi, Carosena Meola

**Affiliations:** 1 Dipartimento Scienze Fisiche, University of Naples "Federico II", Via Cintia, 80134 Napoli, Italy; 2 INFN - Istituto Nazionale di Fisica Nucleare, sezione di Napoli, Napoli, Italy; 3 CNR-SPIN Institute for Superconductors, Oxides and other Innovative materials and Devices, Via Cintia, 80134 Napoli, Italy; 4 CNR-IPCB, Institute for Polymers, Composites and Biomaterials, Viale Kennedy, 54 - Mostra d’Oltremare Pad. 20, 80125 Napoli, Italy,; 5 Department of Industrial Engineering, University of Naples Federico II, Naples, Via Claudio 21, 80125 Napoli, Italy

**Keywords:** graphene, graphite, IR thermography, micro-Raman spectroscopy, strain sensor

## Abstract

In this work we present a novel route to produce a graphene-based film on a polymer substrate. A transparent graphite colloidal suspension was applied to a slat of poly(methyl methacrylate) (PMMA). The good adhesion to the PMMA surface, combined with the shear stress, allows a uniform and continuous spreading of the graphite nanocrystals, resulting in a very uniform graphene multilayer coating on the substrate surface. The fabrication process is simple and yields thin coatings characterized by high optical transparency and large electrical piezoresitivity. Such properties envisage potential applications of this polymer-supported coating for use in strain sensing. The electrical and mechanical properties of these PMMA/graphene coatings were characterized by bending tests. The electrical transport was investigated as a function of the applied stress. The structural and strain properties of the polymer composite material were studied under stress by infrared thermography and micro-Raman spectroscopy.

## Introduction

Many materials have been proposed for strain sensing applications including metals, silicon, carbon nanotubes and graphene. The unique thermal, mechanical and electrical properties of graphene [1] have inspired new and appealing applications in different fields. Its exceptional mechanical robustness and ability to withstand elastic strain (up to 25% for tensile in-plane stretching) make this material interesting for use in advanced technological systems subjected to critical stresses and operating in harsh environments. The large piezoresitivity of graphene is attributed to the charge tunneling mechanism occurring between neighboring platelets, and it critically depends on the composition, density and interconnection properties of the graphene components [2]. Polymers have been used as substrates to support strips of strain sensing components to make a flexible strain gauge. These compounds have received significant interest not only for their high sensibility and tunability, but also for the potential for gauging strain that they offer in several biological systems. A highly stretchable and sensitive strain sensor based on reduced graphene oxide or graphene on polymer were also recently reported [3].

In this work we describe a simple and direct fabrication process based on a new micrographite colloidal suspension to produce thin coatings with large electrical piezoresitivity. When applied to a slat of poly(methyl methacrylate) (PMMA), the adhesion to the PMMA surface, combined with the shear stress, allowed for the uniform and continuous spreading of the graphite nanocrystals on the substrate surface with formation of a very uniform graphene multilayer coating.

The electrical response to the mechanical deformation of a strain sensor is generally quantified by the gauge factor (GF), that is, the ratio of the relative change in electrical resistance, *R*, to the mechanical strain, ε, namely the GF is given as Δ*R*/(ε*R*_0_), where *R*_0_ is the unstrained resistance. The values of GF for graphene range from 1.9–2.4 [2–3]. The GF has been considerably improved by using instead of single graphene layers, self-assembled arrays of nanostructures based on graphene, such as carbon nanotubes [4–5] or graphene nanoplatelets [6–8]. The values of GF on the order of 100 or larger can be obtained in these systems, approaching and in some cases exceeding the value 200, which constitutes the maximum achievable performance of silicon-based sensors.

The electrical resistance and mechanical properties of the graphene-coated PMMA slat were investigated by current–voltage (*I*–*V*) measurements and micro-Raman spectroscopy (μ-RS) during bending tests. Moreover, infrared thermographic technique (IRT) was used to assess the sensitivity of the PMMA/graphene slats to bending deformation.

## Experimental

The graphite used for this strain sensor was prepared according to [9–10] as follows. Expandable graphite flakes (Faima S.r.l., Milano, Italy) were subjected to a thermal shock at 750 °C for 3 min in a muffle furnace to produce expanded graphite. The expanded graphite filaments were converted to nanostructured graphite by ultrasonic treatment in acetone, using a horn sonicator (Bandelin Sonopuls, model UW2200, 20 kHz, 200 W, Berlin, Germany). The suspension (800 mL) was sonicated for 30 min in a glass cylindrical beaker at room temperature (the beaker was placed in a refrigeration bath). The final product was a concentrated colloidal suspension (the nanostructured graphite concentration in this paste was ≈33 g/dm^3^), which was dried in air at room temperature to give the nanostructured graphite powder. Then a concentrated nanostructured graphite colloid was prepared by accurately dispersing the graphite in ethanol (Sigma-Aldrich, 99.9%) under sonication. The nanostructured graphite colloid was gently rubbed on the surface of a slat of PMMA, by hand, using a low-density polyethylene (LDPE) film to achieve a very uniform coating of the substrate surface. The adhesion to the PMMA surface, combined with the applied shear stress, allowed a uniform and continuous spreading of the graphite nanocrystals on the substrate surface with formation of a very uniform graphene multilayer coating. The obtained PMMA slats coated by this very thin and optically transparent layer of graphene were rinsed in acetone and dried in air at room temperature.

**Figure 1:**
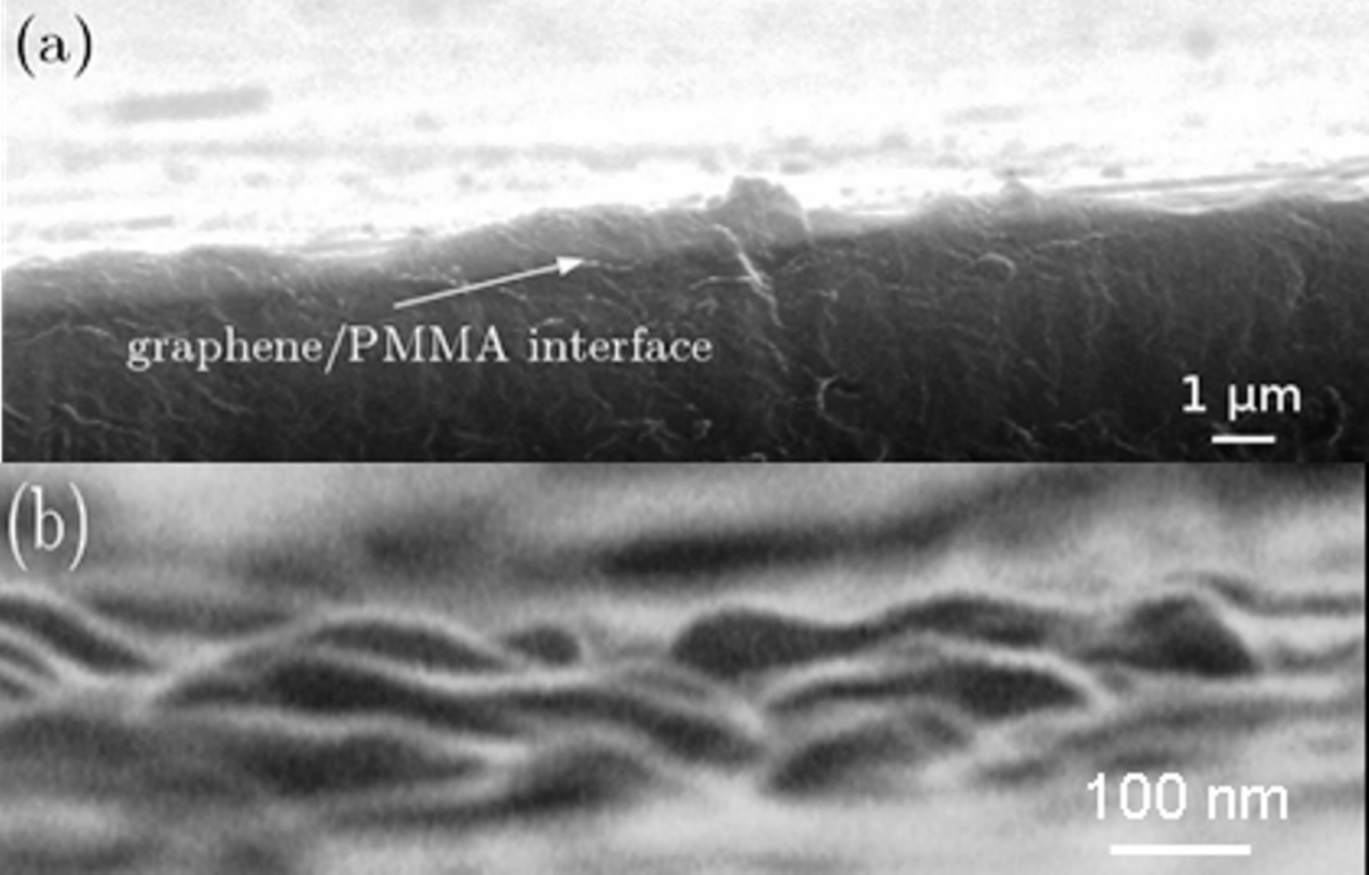
(a) SEM images of the slat section of the graphene/PMMA interface; (b) SEM image of the graphene surface at high magnification.

The graphene compound layer on the top of the PMMA surface is clearly visible in the images of Figure 1, obtained by scanning electron microscopy (SEM) of the slat section. A 0.5 μm thick layer drapes over the PMMA surface (Figure 1a). At high magnification, the granular nature of the coating is evinced from the waviness of the slat surface at the neighboring fracture edge (Figure 1b).

**Figure 2:**
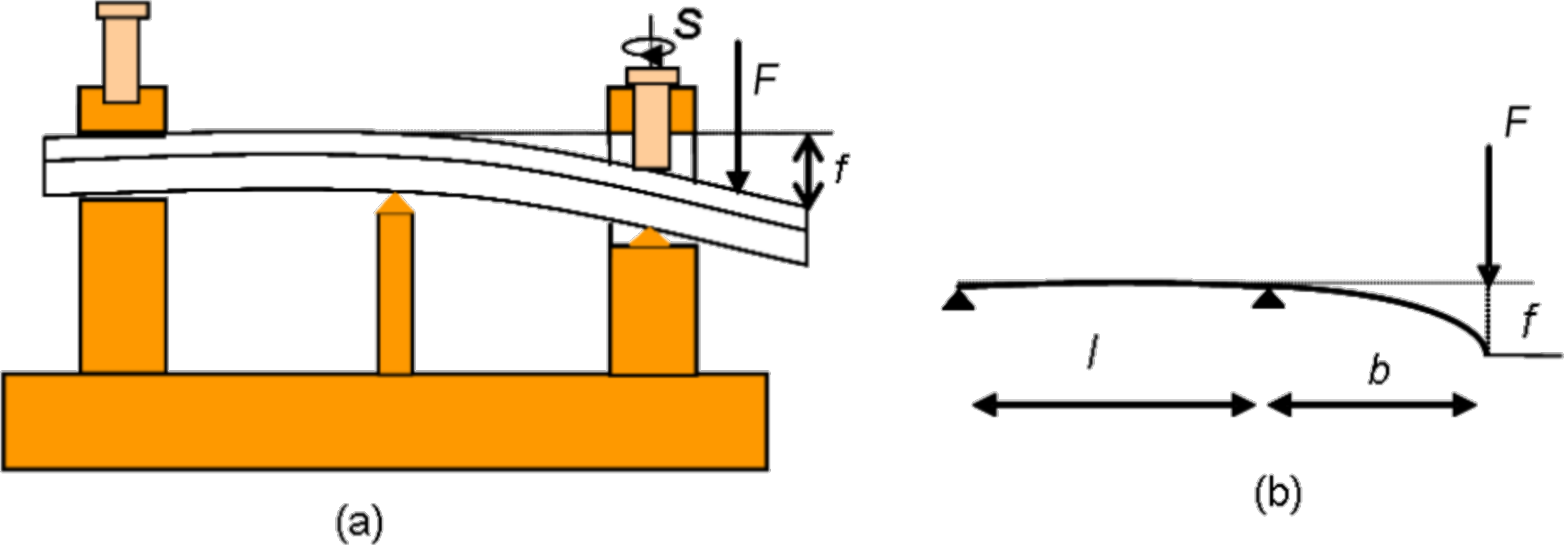
(a) Sketch of the setup used for bending tests; *F* represents the force produced by the screw *S* used to apply the bending stress and *f* is the vertical displacement of the sample surface from the horizontal position. (b) Schematic view of the geometry of the sample under test. The sample dimensions are: 150 × 20 × 3 mm.

The bending stress was applied to the sample by tuning a screw located on one side of the specimen as reported in Figure 2. In this way the quasi-static loading was applied perpendicular to the sample surface as a function of bending. A sketch of the sample under test is reported in Figure 2a.

An estimate of the applied load during the bending test can been obtained by using the follow relation [11]:

[1]
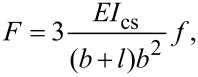


where *f* is the displacement induced by the applied force *F*. *E* is the Young modulus and *I*_cs_ the moment of inertia of the cross section. The lengths *l* and *b* are defined in Figure 2.

In our case a force of 6 *N* is enough to induce a slat deflection *f* = 10 mm, that corresponds to the maximum bending of the slat during the experiment. The force *F* was estimated by assuming *E* = 3.8 GPa for the Young’s modulus value of PMMA [12], and a moment of inertia *I*_cs_ = *wh*^3^/12, where *w* = 0.02 m and *h* = 3 × 10 ^−3^ m are the width and thickness of PMMA/graphene sample, respectively, and *l* = *b* = 75 mm.

The upside surface of the PMMA/graphene material was monitored with an infrared camera (Flir ThermaCam, SC6000), equipped with a quantum well infrared photon detector working in the 8–9 μm infrared band cooled at 70 K by a Stirling cooler, a noise equivalent differential temperature <35 mK, spatial resolution of 640 × 512 pixels (full frame) with pixel size 25 × 25 μm. In the present application, thermal images in time sequence at 60 Hz were collected and after were subjected to postprocessing procedures [13].

**Figure 3:**
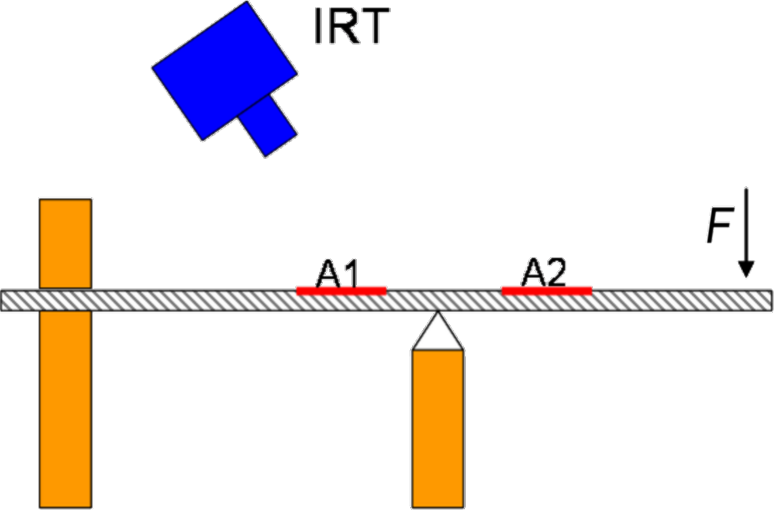
Sketch of the setup used for the thermographic analysis of the PMMA/nanocomposite sample.

Firstly, to account for thermal changes with respect to the ambient temperature, the first image (*t* = 0) of the sequence (i.e., the temperature of the unloaded specimen surface) was subtracted from each subsequent image in order to determine a map of temperature variations:

[2]
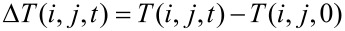


where *i* and *j* are the pixel indices (1 ≤ *i* ≤ 240; 1 ≤ *j* ≤ 320). Therefore, a time sequence of Δ*T*(*i,j,t*) images are created. These images are further analyzed to bring out information on the temperature variations along the specimen surface and to provide structural analysis of the material under investigation. Infrared thermographic tests (IRT) were carried out on the PMMA/graphene coatings and compared to the IRT measurements performed on the various composite polymer-based systems. A sketch of the setup for the thermographic measurements is shown in Figure 3.

**Figure 4:**
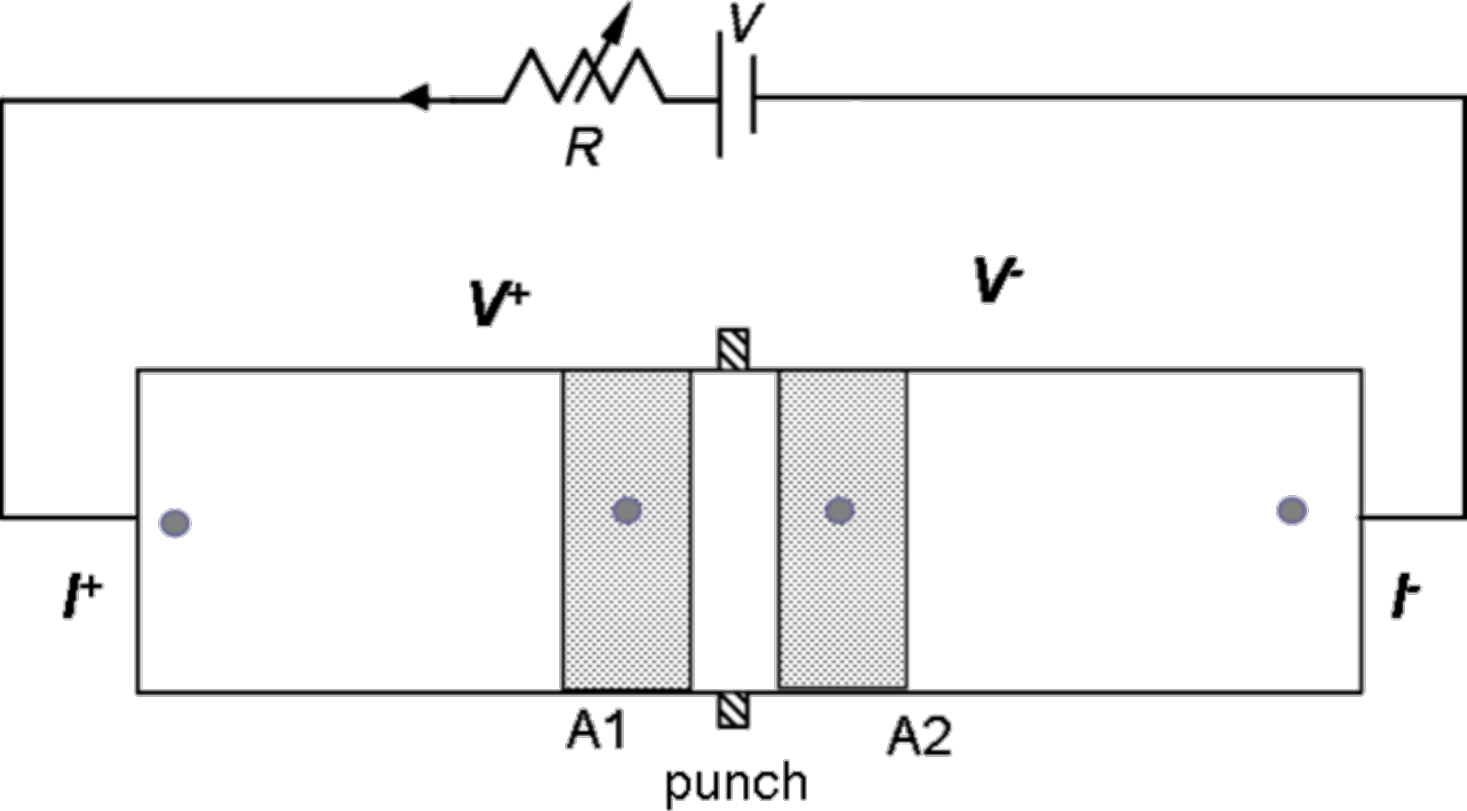
Sketch of the top view of the sample; A1, A2 are the (10 × 10 mm) areas on the sample monitored by the infrared camera during the bending tests. The positions of the voltage (*V*^+^, *V*^−^) and current (*I*^+^, *I*^−^) probes are indicated.

The composites under test include: glass-reinforced plastic (GRP), nanocomposites, and PMMA. The nanocomposite sample is made using an epoxy resin EC01 and filled with multiwalled carbon nanotubes (MWCNT) provided by Nanocyl with a purity grade >95% with an average diameter ≈10 nm and an average length of 1.5 μm. Further details on the nanocomposite fabrication are reported elsewhere [14–15]. The electric transport measurements have been carried out under unload and maximum load conditions. For this purpose a two-probe configuration based on a picoammeter (Keithley, 6487) has been used to measure the current−voltage (*I−V*) curves between the electrodes located on A1 and A2 areas, as shown in Figure 4.

The same setup was used to monitor time changes of the current for *F* = 0 and *F* = 6.9 N. Structural deformation of graphene induced by bending the PMMA slat has been investigated by means of micro-Raman Spectroscopy (μ-RS). The μ-RS measurements were performed by using a Jobin-Yvon system from Horiba ISA, with a TriAx 180 monochromater, equipped with a liquid nitrogen-cooled charge-coupled detector. The grating with 1800 grooves/mm allows for a final spectral resolution of 4 cm^−1^. The spectra were recorded in air at room temperature using a 17 mW He–Ne (λ = 632.8 nm) laser source. The laser light was focused to a 10 μm spot size on the samples through an Olympus confocal microscope with a 50× optical objective.

## Results and Discussion

During the bending stress cycles between unload and maximum load conditions, the temperature variation on the sample surface is measured by the IRT technique over a surface area of (10 × 10 mm) as illustrated by A1 and A2 in Figure 3. It can be seen that the maximum change in temperature occurs for the area A1 at the edge of the slat where the force is applied. As already observed in previous work [14], the temperature variation correlates well with strain modifications: the increase or decrease in Δ*T* depends on the sign of strain.

Indeed, using the thermoelastic effect [16–17] under reversible and adiabatic conditions (i.e., in the elastic regime and neglecting heat transfer within the body and to the environment), for isotropic materials, it is possible to relate the temperature change Δ*T* to the amplitude variation of mean applied stress Δσ (pressure) by the simple relation Δ*T* = −Γ*T*_α_σ where *T* is the absolute temperature of the sample, Γ is the material thermoelastic constant, given by Γ = α/ρ*c*_p_ where α is the thermal expansion coefficient, ρ the density and *c*_p_ the specific heat at constant pressure. For graphene we obtain Γ*T*_α_ = 0.19 GPa^−1^ at room temperature (*T*_α_ = 300 K) using the thermal expansion coefficient α = −3 × 10^−6^ K^−1^ [18], ρ = 2.25 × 10^3^ Kg m^−3^ and *c*_p_ = 2.125 × 10^3^ J·kg^−1^·K^−1^ [19]. The negative sign of the thermal expansion coefficient implies that subjecting the graphene slat to positive dilatation (traction) results in heating whereas a compression gives rise to cooling of the sample. The maximum value of temperature change observed (Δ*T* ≈ 0.1 °C) corresponds to a maximum value of applied stress Δσ of about 0.53 GPa. Accordingly, we obtain Δε/Δσ ≈ 0.28% GPa^−1^ which turns out to be about 0.1% higher than the value reported in [18], probably because of the softening of the PMMA/graphene compound with respect to pure graphene.

**Figure 5:**
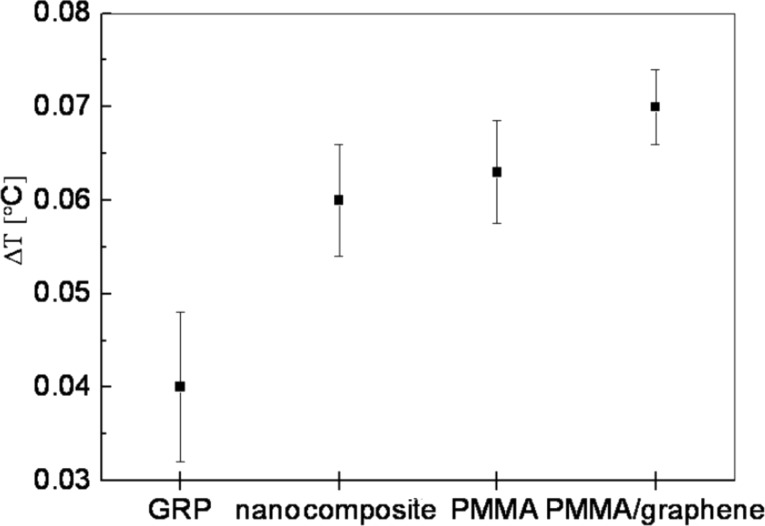
Comparison of the IRT temperature changes. The measurements were performed on various composite materials.

In the Figure 5 the maximum temperature variation Δ*T* for several composites is reported. Here, the different thermal behavior of the new material measured by the IRT technique is shown. The carbon nanotubes (CNTs) can be used as a filler in the polymer composite system to obtain ultralight structural materials (nanocomposites) with enhanced electrical, thermal and optical characteristics. Glass-reinforced plastic (GRP) is used for the realization of the components for use in the automotive and aerospace industry.

Thermographic measurements were carried out across the area A1 (see Figure 3) in the same experimental conditions as those of PMMA/graphene composite. Noticeably, the change in temperature of the graphene/PMMA sample is higher than that of the other considered functional materials, indicating a larger sensitivity of the graphene-based composite in terms of temperature response to external stimulus. In particular, the graphene-coated PMMA substrate exhibits higher temperature changes compared to that of PMMA substrate alone, and a lower thermal noise, as shown in the Figure 5. It is interesting to stress here that this result is obtained on a graphene/PMMA sample where the graphene layer is about 1 μm thick. This behavior can be ascribed to the high thermal conductivity of graphene compared to the other materials reported in Figure 5.

**Figure 6:**
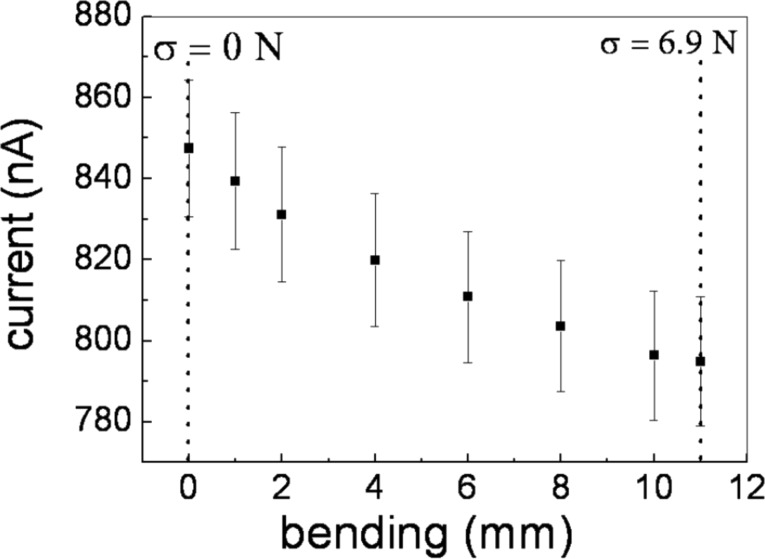
Electrical current variation due to the mechanical stress as measured on a PMMA/graphene sample.

The effect of the bending on the electrical properties of the PMMA/graphene compound were investigated by measuring the current–voltage (*I–V*) using the electrical connection shown Figure 4. The measurement was performed in presence of a voltage of 5 V. Figure 6 reports the results of the current–voltage measurements of the PMMA/graphene sample with and without tensile stress.

**Figure 7:**
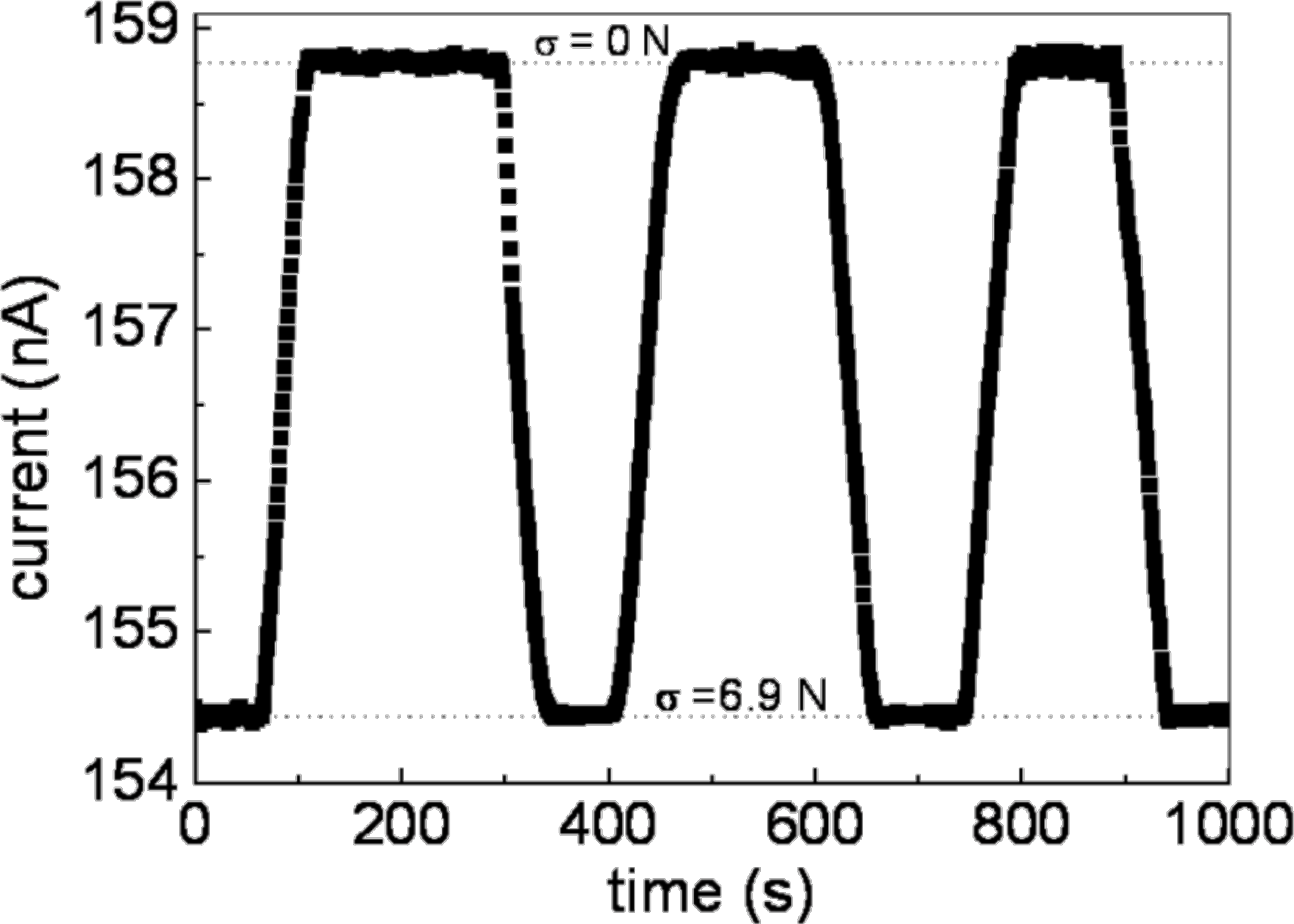
Time dependence of the electrical current for PMMA/graphene. A constant voltage bias *V* = 5 V is applied to the sample while the applied force varies cyclically between unload (*F* = 0 N) and load (*F* = 6.9 N).

Noteworthy, the graphene/PMMA sample exhibits a good Ohmic behavior with a linear increase in electrical resistance to value of about 0.3 MΩ upon application of load up to *F* = 6.9 N. The time dependence of the electrical resistance of the PMMA/graphene sample during loading cycles for a fixed voltage bias *V* = 5 V is shown in Figure 7. The cycle was performed between unload (*F* = 0) and maximum load (*F* = 6.9 N) conditions. Even after several cycles between the unload and loaded state, the electrical resistance value show no hysteretic effect, i.e., the PMMA/graphene does not store stress and the maximum load implemented can be considered as a reversible stress condition.

**Figure 8:**
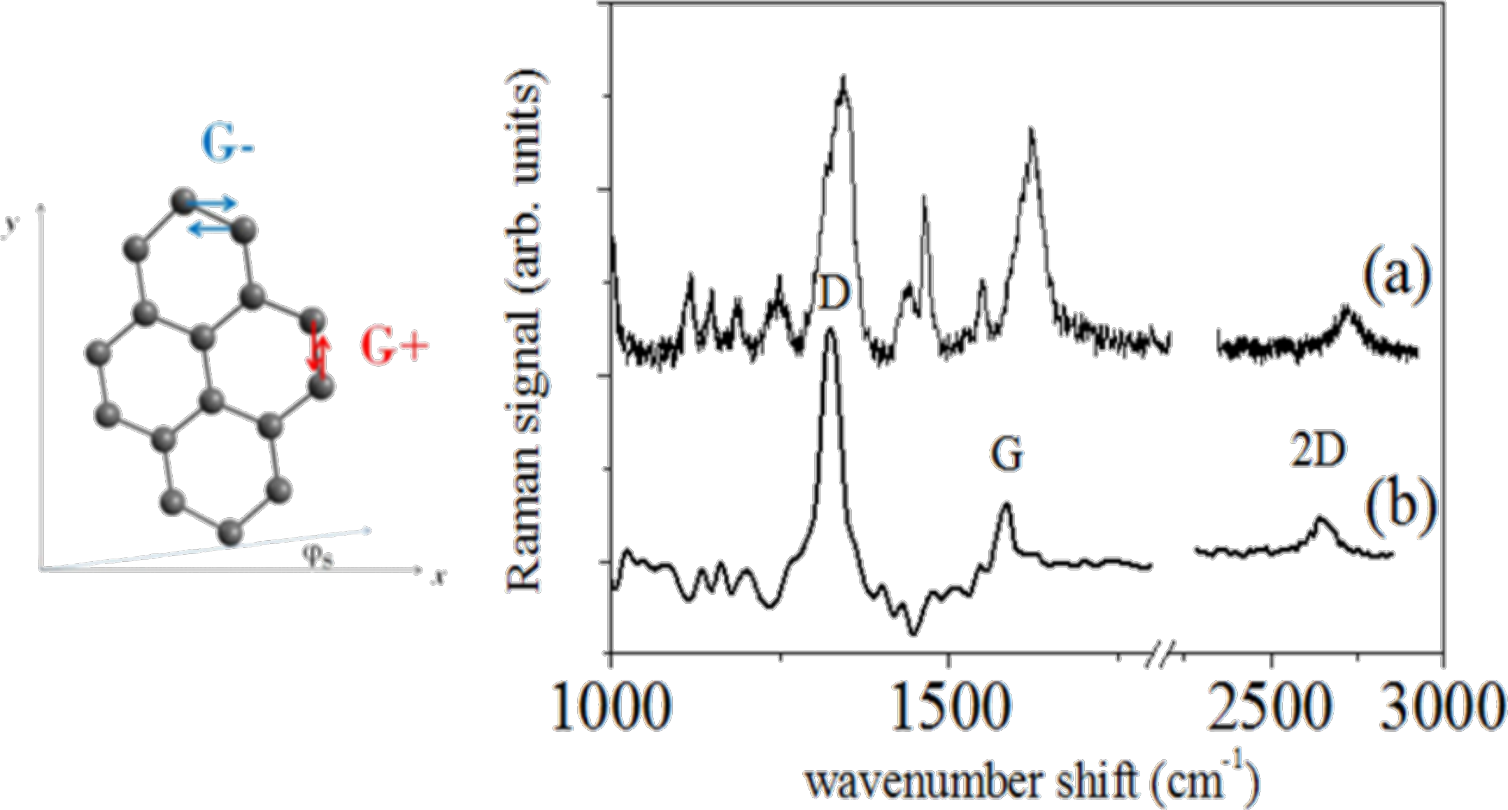
Schematic view of strain induced degenerate vibrational modes E_2g_ shown on the left. The uniaxial strain is directed along the *x* axis. The graphene lattice is turned with respect to the *x* axis at angle φ_S_. On the right, the Raman spectrum of the PMMA/graphene sample is shown as measured (a) and after subtraction of PMMA Raman components (b).

In order to evaluate the microscopic effect of applied stress on sample micro-Raman spectroscopy (μ-RS) has been performed in the region A2 (see Figure 4) during the bending test. The Raman spectrum of the PMMA/graphene structure at rest (i.e., without applied force) is displayed in Figure 8. The Raman spectrum exhibits many peaks, mainly due to the presence of the polymer substrate. The Raman components due to polymer are removed (Figure 8b) by comparing the data with the spectrum acquired on the bare substrate using a numerical data treatment based on a wavelets algorithm and linear regression [19].

**Figure 9:**
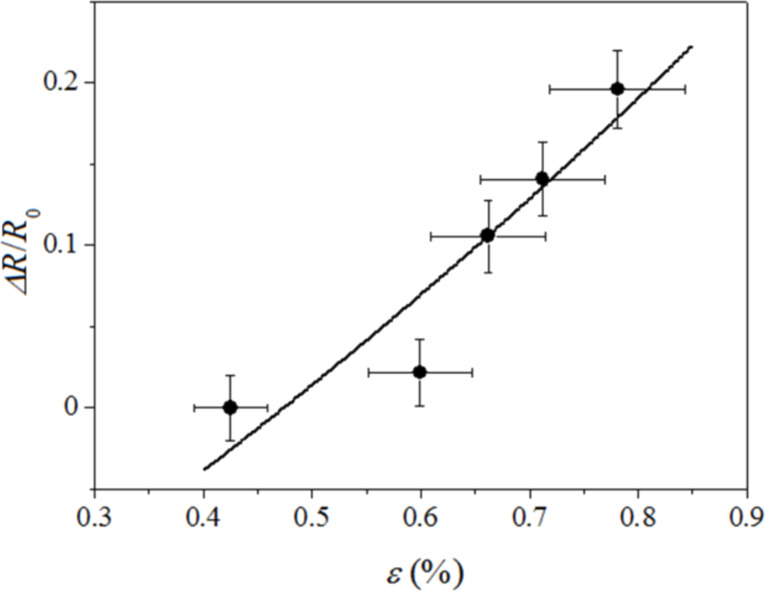
Dependence of the normalized electric resistance variation Δ*R*/*R*_0_ on strain ε. The bars indicate the relative measurement error estimated at 1% and 8% for ε and Δ*R*/*R*_0_, respectively. The solid line shows the best fit to the experimental data as calculated from the theoretical prediction given by Equation 3 with τ = 53 ± 11.

The relative variation of electrical resistance Δ*R*/*R*_0_ is plotted as a function of the applied strain ε (Figure 9), where the data refer to two sets of bending tests. The strain values measured by μ-RS are consistent with those obtained by the IRT analysis, as their variation range is of the order of 0.15%. Noticeable in Figure 9 is that a significant strain, on the order of 0.45%, affects the graphene lattice for the equilibrium position of the slat (no bending) due to intramolecular forces occurring between graphene and PMMA substrate. The GF results were on the order of 40– 50. Note that this value is too high to account for the observed resistance change solely in terms of electronic modifications of the graphene itself [3].

An intergrain electrical transport mechanism should be taken into account, yielding a dependence of the conductivity on the configuration of overlap area and contact resistance of the platelets. The graphene-based layer can be considered as a granular system consisting of metal grains embedded in an insulating matrix and its electrical conduction properties can be described assuming a simple electron hopping mechanism [20]. In this case the dependence of the electrical resistance on the strain can be expressed by the simple relation:

[3]
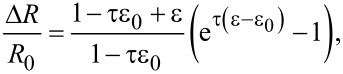


where τ is a parameter proportional to the tunnel barrier depth, *d*, between graphene platelets, *R*_0_ is the value of the electric resistance in unstrained conditions and ε_0_ is the residual strain occurring in the unload sample when resistivity value *R*_0_ is measured.

The data reported in Figure 9 have been fit with Equation 3 using τ as the fit parameter, resulting in τ = 53 ± 11. The parameter τ is proportional to the square root of the actual energy barrier *E*_c_ between grains, which can be approximated by the energy of an electron transfer between a charged platelet to a neutral one as:

[4]
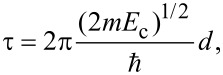


where 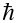 is the Plank constant and the energy barrier *E*_c_ is given by

[5]
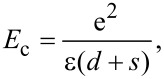


where the average grain size is *s* and ε is the effective relative dielectric constant of the layer. The value of τ found by the fitting procedure implies that for *E*_c_ ≈ 100 meV, an intergrain distance *d* of few nanometers is found, consistently with the adopted model.

From Equation 3, and taking into account the definition of gauge factor, it can be easily see that the GF is approximately 50. Due to this value of the GF, we can conclude that in a microscopic level, a large value of the intergrain separation distance results in improving the sensitivity of the polymer/graphene supported strain sensor.

## Conclusion

In this work we have described a simple fabrication process to produce a low-cost graphene film on a PMMA substrate. The process makes use of the direct application of a nanostructured graphite colloidal suspension to a PMMA slat. Bending tests have been performed on this structure in order to study its piezoelectric response. Electrical, infrared thermography and micro-Raman spectroscopy have been carried out in order to investigate the behavior of the composite system as a local strain sensor. The experiments demonstrated the high piezoresitivity of the system. The electrical resistance changes with applied stress with a gauge factor on the order of 50. The electrical features are consistent with an intergrain electrical transport mechanism among graphene platelets undergoing strain solicitations. The tensile strain of the PMMA/graphene structure during the bending test was confirmed by IRT and μ-RS measurements. The strain magnitude was evaluated by the wavenumber split of the Raman G-mode. The system was modelled as a granular system consisting of metal grains (nanoplatelets) embedded in an insulating matrix and its electrical conduction properties were analyzed within the framework of a simple electron hopping mechanism. The high sensitivity in addition to high stability of the graphene/PMMA structure and the high thermal sensitivity in the infrared wavelength region are interesting aspects that enable us to propose this polymer/graphene supported coating as a new kind of stress sensor useful for applications in different fields from civil, to automotive and aerospace engineering.
